# Antioxidant, antitumor and antimicrobial activities of *Pisolithus albus* extract against some pathogenic bacteria and fungi^☆^

**DOI:** 10.1016/j.jgeb.2026.100813

**Published:** 2026-09-11

**Authors:** Sahar M. Ouda, G.A. Mohamed

**Affiliations:** Plant Research Department, Nuclear Research Center, Egyptian Atomic Energy Authority, 13759, Egypt

**Keywords:** *Pisolithus albus*, Antibacterial, Antifungal, HPLC chromatography, Antioxidant, Cytotoxicity, Antitumor, Heavy metals, Neutron activation technique

## Abstract

**Background:**

*Pisolithus albus* is an ectomycorrhizal fungal species that commonly associates with trees, particularly in loose, sandy soil environments.

**Methods and Results:**

In this study it was collected, molecular identified as new record to Egypt with GenBank accession numbers, PX788599. Then the study focused on determination of capacity of the fungus to produce bioactive substances with antioxidant, antimicrobial and anti-tumor activities. Analysis of the bioactive compound and cytotoxicity were determined. In addition, mineral composition and heavy metal were detected by neutron activation technique (NAA). Fruiting bodies of *Pisolithus albus* were extracted using five various solvents; ethanol, methanol, acetone, ethyl acetate and hexane. Results showed that, *Pisolithus albus* extracts exhibited different antibacterial activities alongside two Gram-postive bacteria *Staphylococcus aureus ATCC6538* and *Bacillus subtilis ATCC6633* and three Gram-negative bacteria, *Escherichia coli* ATCC10536, *Salmonella typhimurium* ATCC 14028 and *Pseudomonas aeruginosa* ATCC9027. Ethanol extract measured the highest antifungal activities against the tested fungal strain; *Candida albicans ATCC10231*. Total phenolic and flavnoids contents were measured. HPLC chromatography of ethanol extracts was performed to investigate the quality of polyphenols and flavonoids. Antioxidant activity of ethanol extract showed considerable DPPH radical scavenging activities, which exhibited higher DPPH radical inhibition (94.71%) compared with standard (98.65%).Also ethanol extract was tested against three cancer cell line Hep G-2 cells (human Hepatocellular cancer cell line, MCF-7 cells (human breast cancer cell lines, A-549 (Lung cancer cells).

**Conclusions:**

Collectively, these findings position *Pisolithus albus* as a valuable and versatile biotechnological resource bioactive compound with potential therapeutic applications.

## 1 Introduction

*Pisolithus* is an ectomycorrhizal fungus commonly associated with trees growing in loose, sandy soils, especially along coastal pine woodlands. These fungi can grow quite vigorously and even through surfaces covered with asphalt, the genus *Pisolithus* is from Greece, piso (pea) and lith (stone).^1^ These genera are found in a wide variety of environments with different temperature, nutrient and heavy metal levels, and have been reported in temperate, tropical and subtropical regions.^2^, ^3^, ^4^

Martin et al. (2002)^5^ recognize three definite evolutionary lineages in *Pisolithus,* comprising at least 11 species. More recent records indicate that the genus currently includes 20 recognized species (MycoBank 2022). Of these, *Pisolithus albus* is a widely distributed taxon, reported from several countries of the world such as Australia, China, India, Malta, Brazil, Morocco, Italy, New Zealand, New Caledonia, Senegal, Thailand, Spain, Tunisia, Pakistan and USA. However, this occurrence is new report of *P. albus* in Egypt. *P. albus* may be divided into four genetically distinct ecotypes.

The genetic differentiation and geographic distribution models may be associated with long-range dispersal events and may also have played a role in explaining the species remarkable mycorrhizal colonization of a variety of hosts.^6^, ^7^ Macrofungi are important nutritional resources and traditional remedies since ancient times, especially in Japan and China. In the past few decades, many bioactive metabolites have been extracted from basidiomycota that exhibit antibacterial, antifungal, and antiviral activity.

The *Pisolithus* spp. are widespread around the world in temperate and tropical regions and form ectomycorrhizal associations with numerous woody plants, but there is little information on their antimicrobial activity. Their symbiotic association with host plants, genetic diversity, taxonomy, management practices, soil improvement and erosion control have been the topics of the previous studies.^8^, ^9^ The current study was undertaken to assess the antioxidant, antimicrobial and antitumor activities of *Pisolithus* extracts in order to evaluate the potential of using them as therapeutic candidates.

## 2 Materials and methods

### 2.1 Sample collections and morphological description

A sandy soil macro-fungus was wild located around the area of Ismailia Canal Zone, El Qulubia Governorate, Egypt. It was collected at various intervals throughout the period among September 2025 and November 2025. Collected samples were situated in sterile plastic bags and carried in the lab for further analysis and examination. The fungus was cleaned, and the specimens were harvested and the genus identified, comparing and contrasting the microscopic as well as the macroscopic observations made with the known species, and identifying the genus to be the same one as described by O'Reilly.^1^

### 2.2 Molecular identification and Phylogenetic tree analysis of selected isolate

Genomic DNA was isolated directly from fungal fruiting bodies utilizing the QIAampDNeasy Plant Mini Kit (Cat. No. 69104) due to the manufacturer's instructions. ITS1/ITS4 was the primer set used to amplify ITS region.^10^ The PCR products were initially purified on a QIAquick PCR Purification Kit (Qiagen, Valencia). Then, the cycle sequencing was carried out with the BigDye Terminator v3.1 Kit (Perkin-Elmer). These sequencing reactions were then further purified by using Centri-Sep spin columns. Lastly, sequencing was performed on an Applied Biosystems 3130 Genetic Analyzer (HITACHI, Japan). The initial sequence annotation was done by comparing the sequences to GenBank sequences using the NCBI BLAST® platform.^11^ Sequences were also compared using the Lasergene DNASTAR v12.1 software.^12^ Next, phylogenetic reconstruction was carried out in the software MEGA6 using the neighbor-joining method with the Utmostcombined likelihood model.^13^

### 2.3 Preparation of Pisolithus albus extracts

A sandy soil fungus *Pisolithus albus* was collected. The fresh fungus was cleaned and dried. The dried *Pisolithus* material was milled into a fine powder and kept in a dark, dry environment at room temperature until extraction. Separately, 40 g of the powder was mixed with 200 ml of each solvent (ethanol, methanol, acetone, ethyl acetate, and hexane) and stirred for 2 h at ambient temperature. After collecting the supernatants, the solvents were eliminated using a rotary evaporator, and the resulting extracts were preserved at 4 °C for subsequent analyses.

### 2.4 Antimicrobial activity of Pisolithus albus

The antibacterial activity of *Pisolithus albus* for ethanol, methanol, acetone, ethylacetate and hexane extracts were tested against five pathogenic bacteria strain. Gram positive bacterial strain (*Staphylococcus aureus ATCC6538* and *Bacillus subtilis ATCC6633*, Gram-negative bacteria (*Escherichia coli* Strain ATCC10536, *Salmonella typhimurium* stain ATCC 14028, *Pseudomonas aeruginosa* strain ATCC9027), and *Candida albicans ATCC10231*. The bacterial strains and yeast were obtained from microbiology Lab, Arab Company for Pharmaceuticals and Medicinal Plants (Mepaco), Sharkiya, Egypt. All tested strains were maintained on nutritive agar and stored at 4 °C.The antimicrobial potential of five solvent-derived extracts was evaluated utilizing the agar well diffusion assay by determining the inhibition zones (mm) against pathogenic microorganisms. Wells (10 mm in diameter) were cut using a sterile cork borer from agar media and 100 μL of each extract was added into the wells in inoculated plates. Bacterial and yeast isolates were grown at 37 °C for 24 h, while filamentous fungi were protected separately at 29 °C for a interval of 48 h, sterilized distilled water was used as negative control and gentamycin and Ketoconazole were used as positive standard. After incubation inhibition zone diameters were measured and minimum inhibitory concentrations (MICs) were determined. All the experiments were repeated three times, and all the measurements were recorded.^14^, ^15^

### 2.5 Total phenolic content (TPC) assay

Total phenolic content (TPC) was measured as described by Folin-Ciocalteu colorimetric method.^16^, ^17^ In brief, 0.05 ml of the ethanolic separate was mixed with 3 ml of 10% Folin–Ciocalteu reagent and 0.8 ml of 7.5% sodium bicarbonate. After 30 min incubation at room temperature the absorbance was measured at 765 nm on a Milton Roy Spectronic 1201 spectrophotometer. The results were conveyed as micrograms of Gallic acid equivalents (μg GAE)/g of extract.

### 2.6 Total flavonoid content (TFC) assay

0.1 ml of extract, 3.9 ml of distilled water, and 0.3 ml of 5% sodium nitrite were added and the mixture was incubated for 5 min. Then 0.3 ml of 10% aluminum chloride was added and incubated for 6 min. After this, 2 ml of 1 mol L^−1^ sodium hydroxide and 2.4 ml of distilled water were added. The absorbance was measured at 510 nm against a non-reacted blank, with the help of a Milton Roy (Spectronic 1201) spectrophotometers. The concentration of quercetin equivalents (QE) was reported as μg QE g^−1^ extract.^18^

### 2.7 HPLC for determination of phenolic and flavonoids compound of ethanol extract

HPLC analysis was performed using an Agilent 1100 HPLC system fitted with dual LC pumps, UV–Vis detector and an Agilent C18 column (125 × 4.6 mm, 5 μm). Agilent ChemStation software was used for collecting and handling chromatographic data. Phenolic acids were quantified with an isocratic mobile phase of 0.1% methanol–phosphoric acid (50:50, *v*/v) at 1.0 ml/min flow rate and at 280 nm. The flavonoids were separated under isocratic conditions with a methanol/water mixture (50/50, *v*/v) at pH 2.8 (adjusted with phosphoric acid) flowing at 1.0 ml/min.^19^

### 2.8 2,2-diphenyl-1-picrylhydrazyl (DPPH) radical scavenging activity

A freshly prepared 0.004% (*w*/*v*) methanolic solution of 2,2-diphenyl-1-picrylhydrazyl (DPPH) was kept in the dark at 10 °C. The test compound was also dissolved in methanol. An aliquot of 40 μL of the sample solution was mixed with 3 ml of the DPPH solution. Absorbance was immediately recorded using a UV–visible spectrophotometer (Milton Roy, Spectronic 1201). The reduction in absorbance at 515 nm was monitored continuously at 1-min intervals until stabilization (16 min). Measurements were also performed for the DPPH control (without antioxidant) and for ascorbic acid as the reference standard. All analyses were performed in three independent replicates, and the average of the obtained values was considered for subsequent evaluation. The percentage of DPPH radical scavenging activity (PI) was calculated according to the following equation:

PI = [{(*A*C- *A*T)/ *A*C} x 100] (1).

Where AC and AT are the absorbance of the control and the absorbance of the sample mixed with DPPH after 16 min, respectively.^20^ Dose–response curves were generated and analyzed using GraphPad Prism software (San Diego, CA, USA) to determine the IC₅₀ (half-maximal inhibitory concentration) – the concentration of an inhibitor should to inhibit DPPH radical scavenging by 50%.

### 2.9 Cytotoxicity and antitumor evaluation

**Mammalian cell lines for Cytotoxicity assay**: The human skin fibroblast cell line HSF-1 and the diploid lung fibroblast line WI-38 were sourced from the American Type Culture Collection (ATCC, Rockville, MD, USA). The experimental assay was conducted at Al-Azhar University.

**Cell line Propagation for Cytotoxicity assay:** Cells were cultured in Dulbecco's Modified Eagle Medium (DMEM) enriched with 10% heat-inactivated fetal bovine serum, 1% l-glutamine, HEPES buffer, and 50 μg/ml gentamycin. Incubation was carried out at 37 °C in a humidified environment encompassing 5% CO₂. The cultures were subcultured twice weekly to maintain optimal growth conditions.

**Mammalian cell lines for antitumor assay:** Human cancer cell lines HepG2 (hepatocellular carcinoma), MCF-7 (breast adenocarcinoma), and A549 (lung carcinoma) were procured from the American Type Culture Collection (ATCC, Rockville, MD, USA). The experimental assay was accompanied at Al-Azhar University.

**Cell line Propagation for antitumor assay:** Cells were cultured in RPMI-1640 medium enriched with 10% heat-inactivated fetal bovine serum and 50 μg/ml gentamicin. Incubation was carried out at 37 °C in a humidified environment containing 5% CO₂. The cultures were routinely subcultured two to three times per week to maintain optimal growing conditions.

Cells were broadcast into Corning® 96-well tissue culture plates at a density of 5 × 10^4^ cells per well in culture medium and incubated for 24 h. Afterwards, the fungal extract was added in triplicate wells to obtain eight different concentrations for each tested compound. For each plate, six wells containing medium only (vehicle control) were included. After an additional 24 h of incubation, cell viability was determined using the 3-(4,5-dimethylthiazol-2-yl)-2,5-diphenyl tetrazolium bromide (MTT) assay. In short, the culture medium was taken out and replaced with 100 μL of new phenol red free medium. Then, 10 μL of 12 mM MTT solution was added to each well (involving the controls). The plates were then left to incubate for 4 h at 37 °C in an atmosphere of 5% CO₂ and humidity. The 85 μL of culture medium was carefully removed from each well and 50 μL of DMSO was added. The mixture was then thoroughly homogenized using a pipette and incubated at 37 °C for 10 min. After that, the absorbance at 590 nm was measured with the microplate reader (SunRise, TECAN Inc., USA) to find out the amount of viable cells.

The cell viability was calculated as a percentage operating the following equation: [(ODt / ODc) × 100%], where ODt is the mean optical density of the treated samples, and ODc is the mean optical density of the untreated control cells. Survival curve of the treated cell line was plotted after exposure to the cell line to the compound under test for varying concentrations of the compound and values of cell viability. The dose–response plots were drawn using GraphPad Prism software (version 6.0; San Diego, CA, USA) and the cytotoxic concentration (CC50) was calculated as the dose that killed 50% of cells.^21^, ^22^, ^23^

### 2.10 Neutron activation analysis (NAA)

The elemental compositions of crude fungal powder of two different isolates collected at different locations were obtained by neutron activation analysis (NAA). These samples were neutron irradiated and the induced gamma radiation captured by a high purity germanium (HPGe) detector. The obtained gamma spectra was then evaluated for the identification of elements and determination of the concentration of the elements. The method used was according to the analytical protocol provided by the Egypt Second Research Reactor (ETRR-2).^24^

### 2.11 Statistical analysis

All information were depicted as mean values ± standard deviation (mean ± S.D.), based on three independent triplicate measurements. Statistical significance between control and treated samples was assessed using one-way analysis of variance (ANOVA). Results were thought significant when *p* < 0.05.

## 3 Results

### 3.1 Morphological description of the fungus

*Pisolithus albus* (as depicted in fig. (1) is a fungus of Phylum: Basidiomycota- Class: Agaricomycetes - Order: Boletales Family: Sclerodermataceae. The fruiting body is generally ellipsoid to ovoid, mostly in the early stages. As it matures, sometimes a brief, simple stipe is developed. It consists of fertile spore-producing tissue (Fig. 1A–C). The basidiocarp is typically about 5–10 cm in diameter, but can sometimes be up to 20 cm along the largest dimension, often vertical. In the early stage, the internal part (peridium) is made up of many separate chambers, which look like compacted grains (pseudoperidioles) in which the spores are formed (Fig. 1D). The walls of these pseudoperidioles are thin and as the fruit matures the spores are released. Eventually the spore dissemination takes place when the outer layer of the peridium bursts and disperses. The spores are globose to subglobose, golden yellow and reddish-brown when mature. Generally, 7 to 11 μm in diameter, and covered in irregular triangular spines up to 2 μm tall (Fig. 1E).

**Fig. 1:**
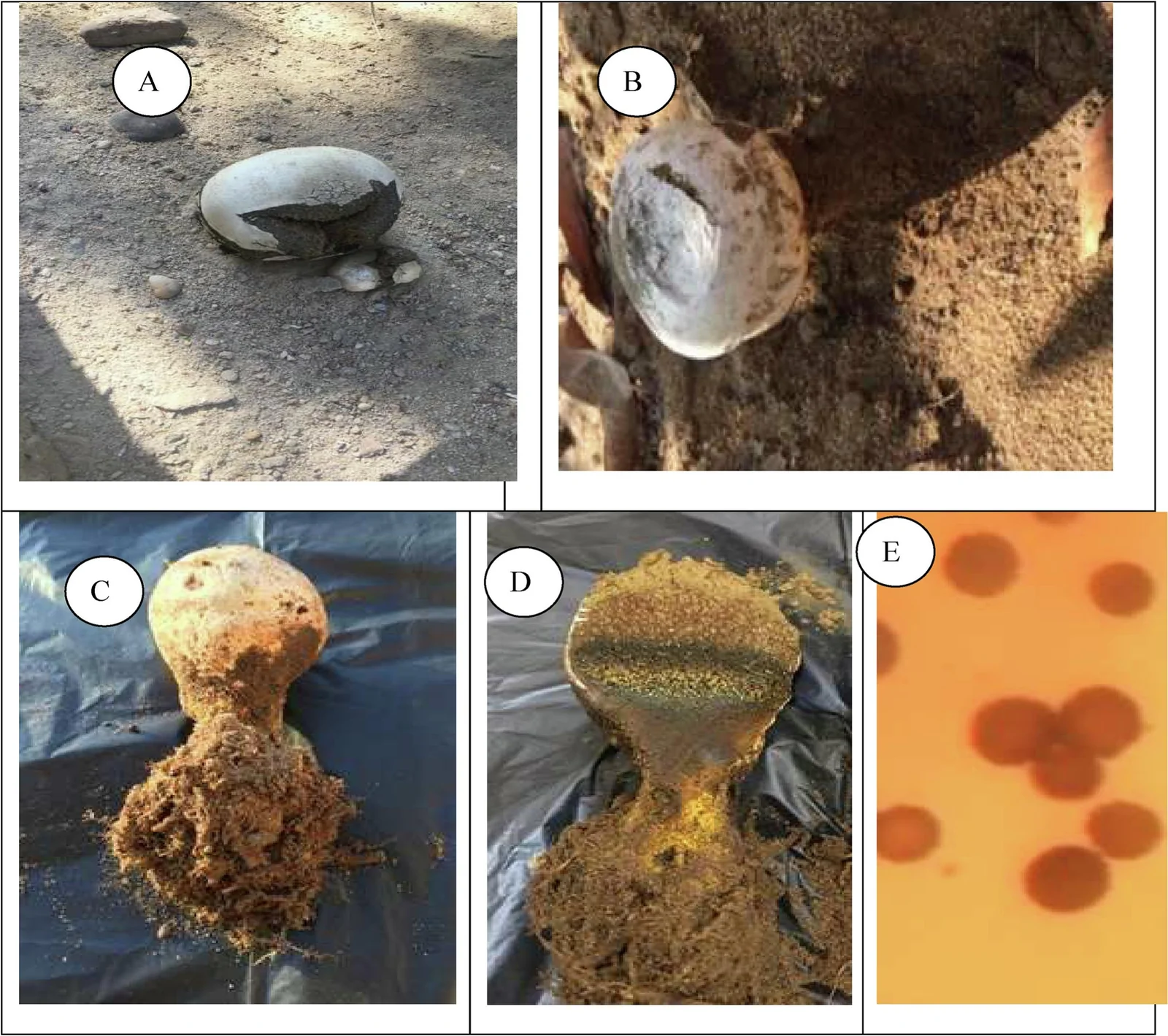
*Pisolithus albus* basidiocarp,A–C; peridium D; basidiospores E.

### 3.2 Molecular identification of Pisolithus albus

The total number of taxa in the combined rDNA dataset from ITS and LSU was 37, of which 12 taxa belonged to *Pisolithus albus* complex and 26 taxa were representatives of other species of *Pisolithus*. Fig. 2. The 18S rRNAgene of fungi was used for phylogenetic relatedness. Maximum-likelihood tree generated after 500 bootstraps designated clustering of tested strain with *P. albus* strains apart from other *Pisolithus* types. Sequence distance of the fungal ITS region of the tested strain (generated by lasergene software) showing 99.5–100% identity of tested strain with *P. albus* strains (Fig. 3.), while it showed 71.2–92.4% identity with other Pisolithus types. Fig. (4) Nucleotide alignment details for all studied strains. Nucleotides alignment of the examined strain showing great homology between tested strained *P. albus* strains.**GenBank accession numbers**.: PX788599. (See Fig. 4, Fig. 5, Fig. 6.)

**Fig. 2:**
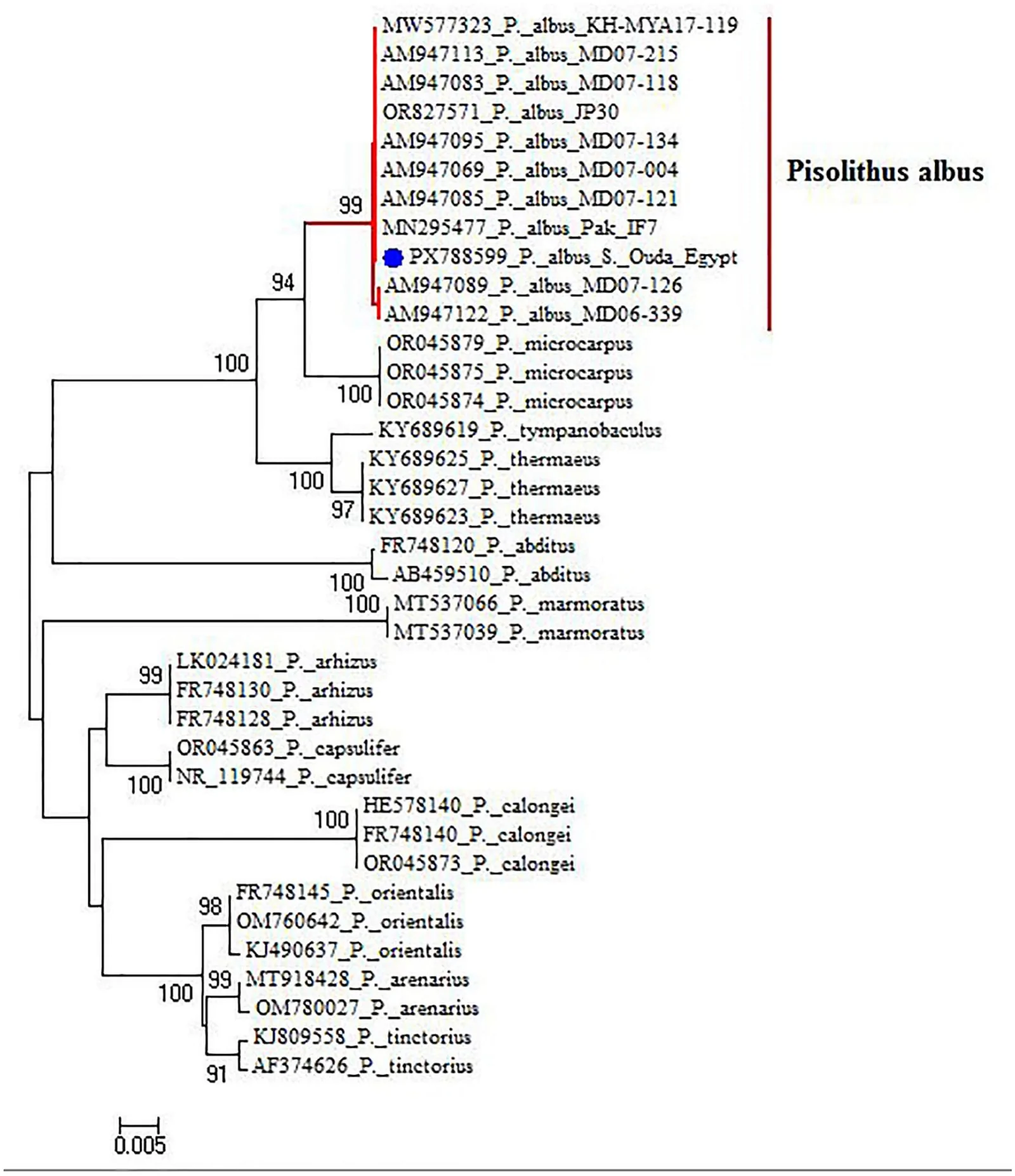
Phylogenetic relatedness of fungal ITS region. Maximum-likelihood tree produced after 500 bootstraps revealed clustering of tested strainwith *P. albus* strains apart from other *Pisolithus* types.

**Fig. 3:**
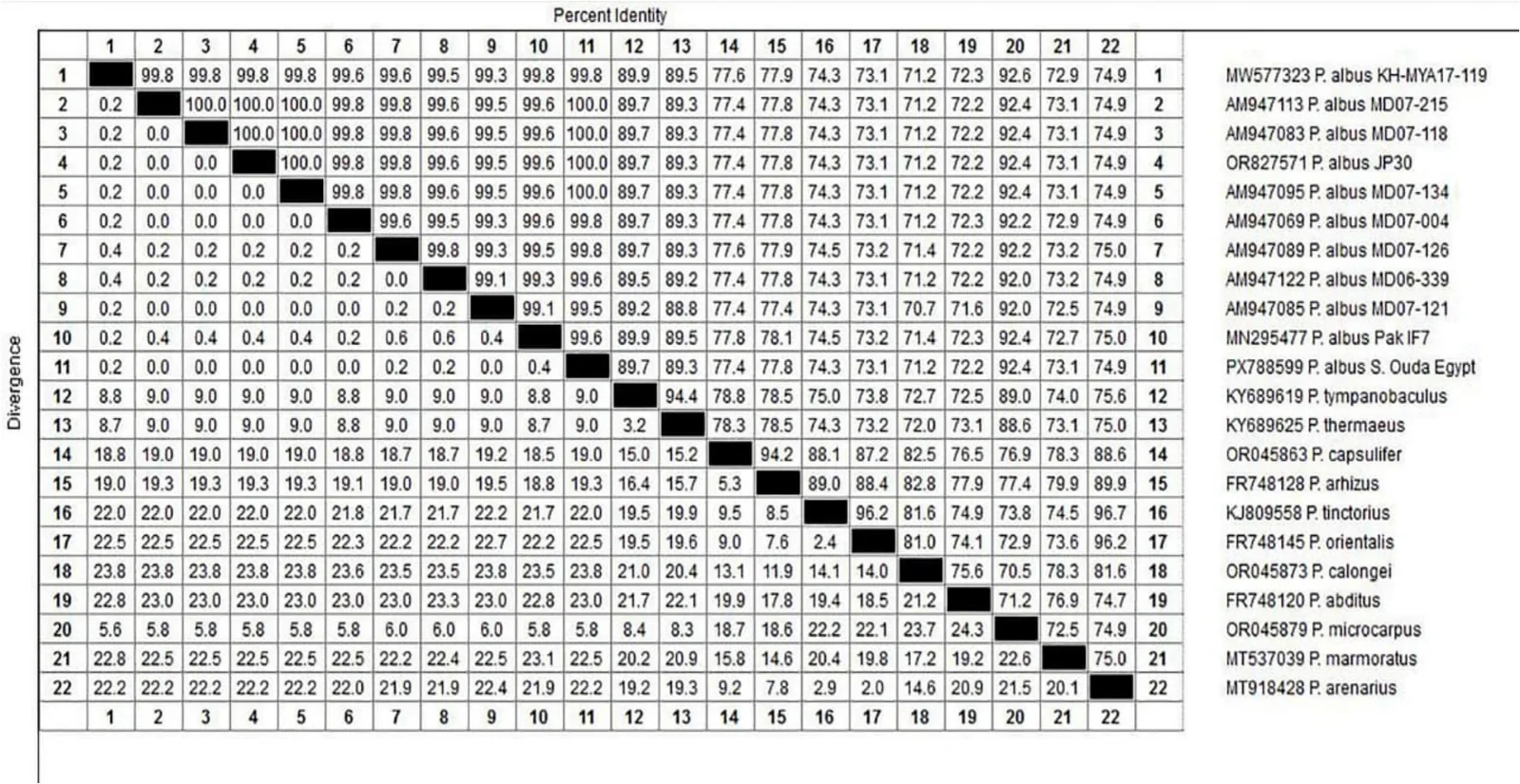
Sequence distance of the fungal ITS region of the tested strain (generated by lasergene software) exhibiting 99.5–100% identityof tested strain with *P. albus*strains, while it showed 71.2–92.4% identity with other *Pisolithus*types.

**Fig. 4:**
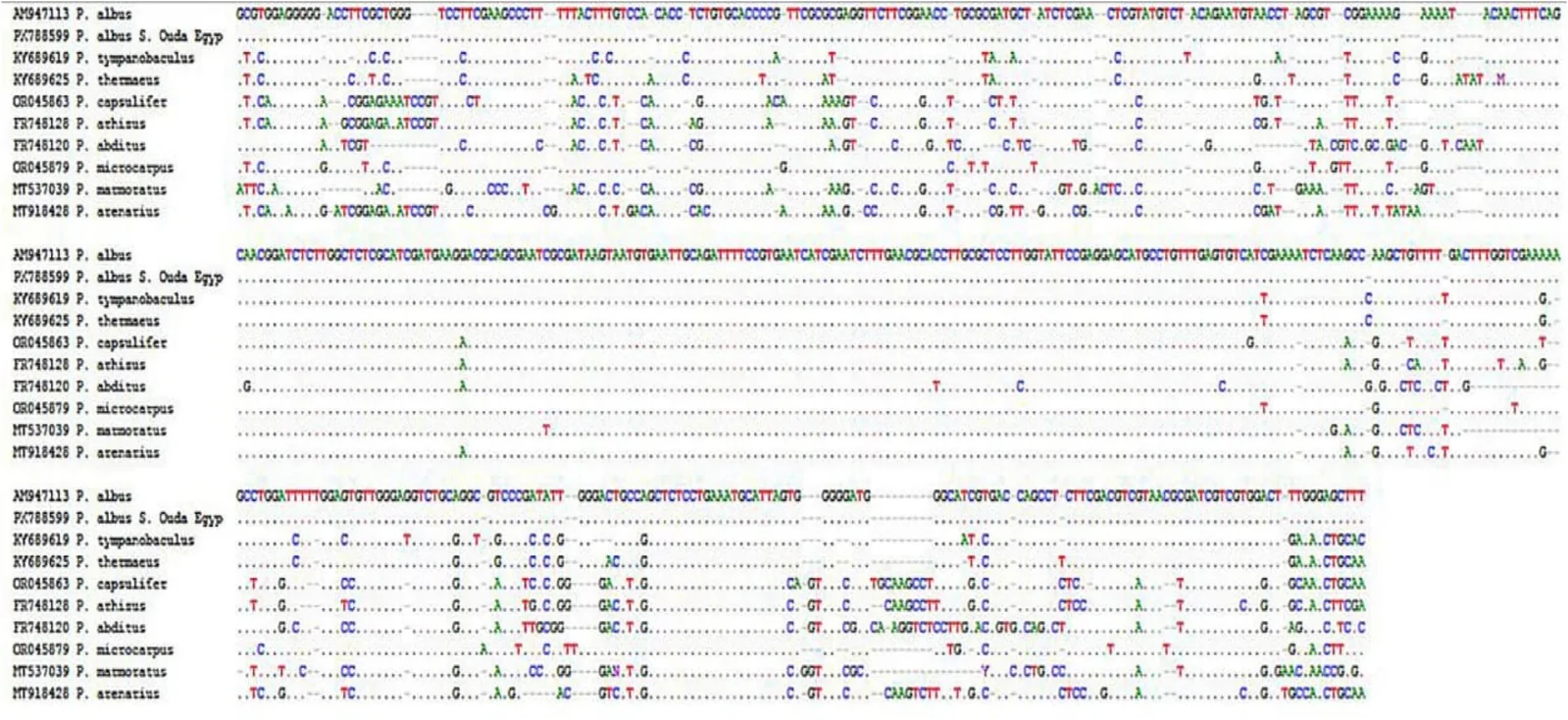
**Nucleotide alignment report for all studied strains.** Nucleotides alignment of the investigated strain illustrating the great homology between tested strain and *P. albus* strains.

**Fig. 5:**
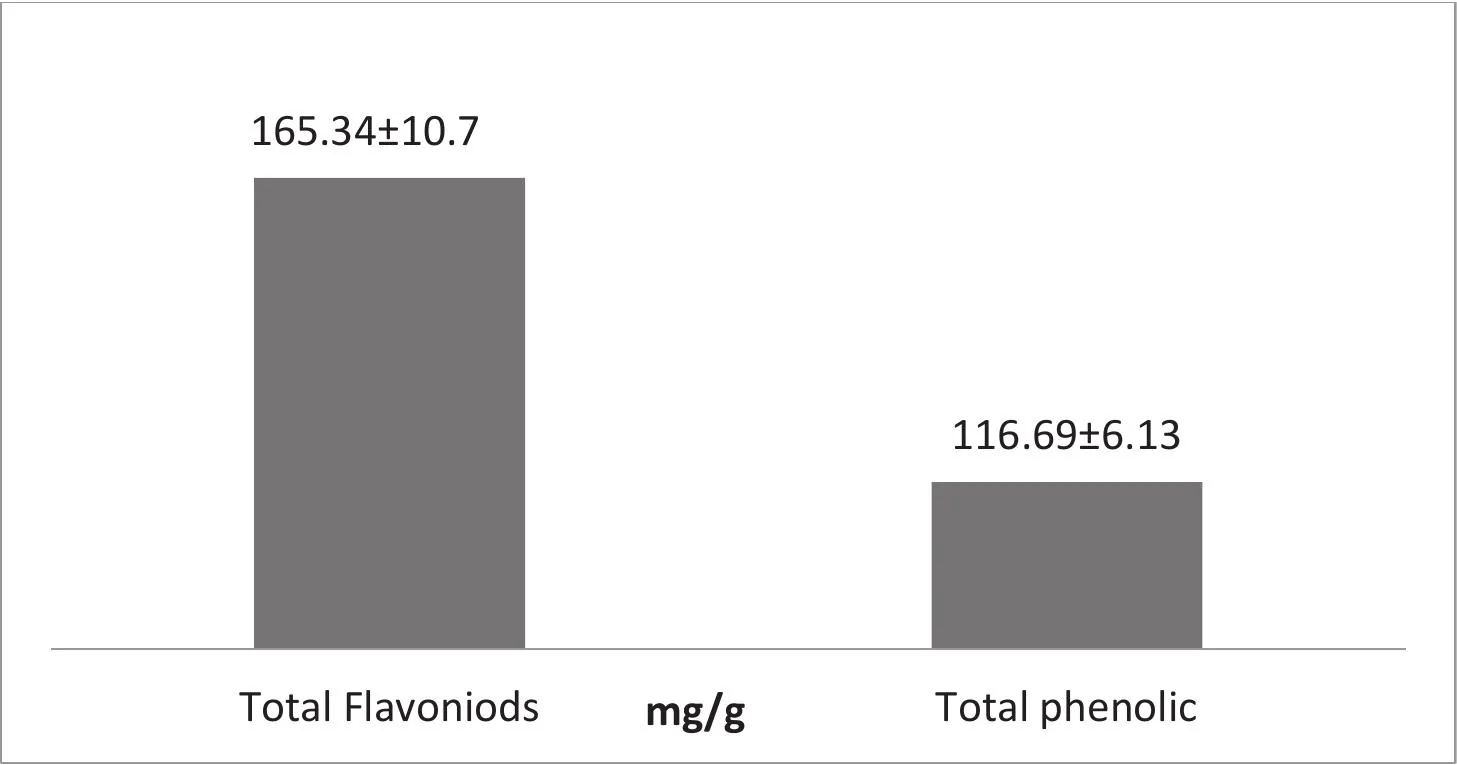
Total Total Phenolic and Flavonoids Content of ethanol extract Gallic acid equivalents, *QE* quercetin equivalents.

**Fig. 6:**
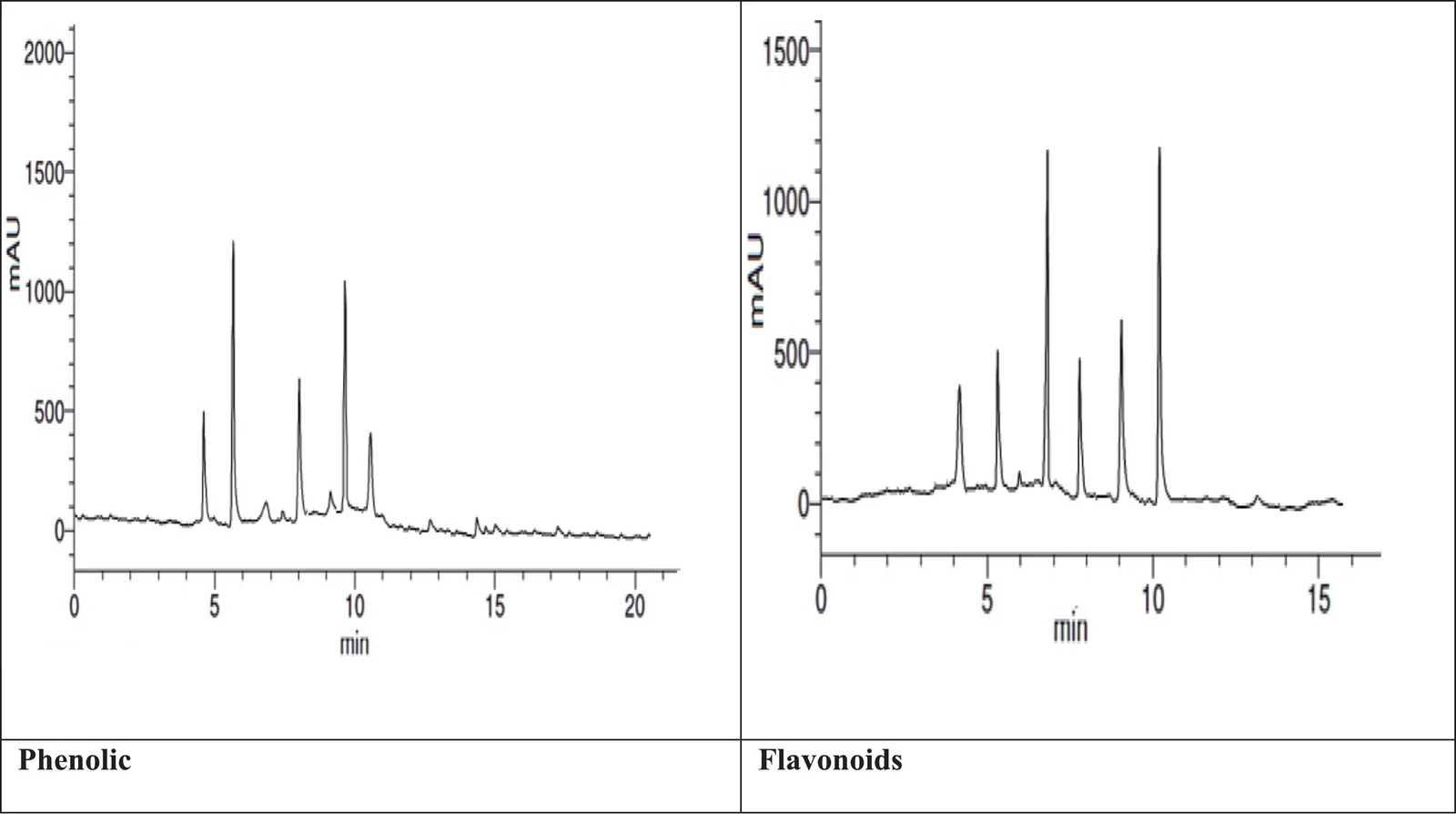
HPLC analysis of Phenolic and Flavonoids compounds of ethanol extract of *Pisolithus albus*.

### 3.3 The antimicrobial activity of Pisolithus albus extracts against the pathogenic bacterial and fungal strains

The antimicrobial potential of *Pisolithus albus* extracts was evaluated in vitro using the agar well diffusion technique against five pathogenic bacterial strains as well as a yeast species. All tested extracts were assessed and the findings are presented in Table 1. The observed clear inhibition zones confirmed that *Pisolithus albus* possesses noticeable antimicrobial activity. The ethanol and acetone extracts of *Pisolithus albus* affected *Salmonella typhimurium*(23.6 mm) *and Bacillus subtilis respectively (*23 mm) followed by *Ps. aeruginosa* (22 mm)*, Bacillus subtilis* (21 mm) and *Staphylococcus aureus* (20.5) by ethyl acetate extract, whereas the *E. coli* low *affected* by all tested solvent extracts (15 mm).Regarding antifungal activity, Ethanol extract affected *Candida albicans* with an inhibition zone (23–18.3 mm) with all tested extracts. MIC for fungal extracts were measured and showed in Table 2. The most efficient extract was ethanolic extract with MIC 20 mg/ml against all tested pathogenic isolates. *Candida albicans* were more sensitive to all tested fungal extracts at MIC20 mg/ml. (See Table 2, Table 3, Table 4.)

**Table 1:** The antimicrobial activity of *Pisolithus albus* extracts against the pathogenic bacterial and fungal strains.

Inhibition zone (mm)						
Fungus extract 50 mg/ml	**Gram-negative bacteria**			**Gram-positive bacteria**		**Yeast**
	*E. coli*	*S. typhimurium*	*Ps. aeruginosa*	*Staphylococcus aureus*	*Bacillus subtilus*	*Candida albicans*
Ethanol	**15** **± 0.5**	**23.6 ±** **0.6**	**19** **±** **0.5**	**16.5** **±** **0.5**	**20** **±** **0.1**	23.3 ± 1.5
Methanol	**15** **±** **0.5**	**15.6** **±** **0.6**	**15** **±** **0.5**	**20** **±** **0.1**	**20** **±** **0.5**	18.3 ± 2.5
Acetone	**15** **±** **0.5**	**22** **±** **0.5**	**20** **±** **0.5**	**17.5** **±** **0.5**	**23** **±** **0.5**	19.5 ± 0.7
Ethyl acetate	**15** **±** **0.5**	**22** **±** **0.5**	**22** **±** **0.5**	**20.5** **±** **0.5**	**21** **±** **0.6**	22.3 ± 1.2
Hexane	NA	NA	NA	NA	NA	NA
Gentamicin	30 ± 1.5	23 ± 1.2	12 ± 0.5	27 ± 1.5	26 ± 1.6	–
Ketoconazole	–	–	–	–	–	21 ± 1.2

NA: No Activity.

**Table 2:** The MIC of *Pisolithus albus* isolates versus the pathogenic bacterial and fungal strains.

Solvent Extract	MIC (mg/ml)					
	*E. coli*	*S. typhimurium*	*Ps. aeruginosa*	*Staphylococcus aureus*	*Bacillus subtilus*	*Candida albicans*
ethanol	**20**	**20**	**20**	**20**	**20**	20
Methanol	50	50	50	50	50	20
Acetone	50	50	50	**20**	**20**	20
Ethyl acetat	50	50	50	**20**	**20**	20
Hexane	NA	NA	NA	NA	NA	NA

NA: No Activity.

**Table 3:** Total Phenolic and FlavonoidsContents of ethanol extract Gallic acid equivalents, *QE* quercetin equivalents.

Ethanolic extract	Total Flavonoids (mg/g)	S.D. (±)	Total Phenolics (mg/g)	S.D. (±)
	165.34	10.7	116.69	6.13

**Table 4:** HPLC analysis of Phenolic and Flavonoids compounds of ethanol extract of *Pisolithus albus*.

	Flavonoids		
Fraction no.	Retention time	Compound	Concentration ug/g
1	4	Naringin	5.40
2	5.2	Rutin	6.26
3	7	Querestin	13.95
4	8	Kampferol	5.12
5	9	Luteolin	8.41
6	10.1	Apegenin	16.54

Phenolic compounds			
1	4.9	Syringenic	4.11
2	6	p-Coumaric	12.52
3	8	Caffeic	6.24
4	9	Pyrogallol	0.54
5	9.8	Gallic	16.75
6	10.8	Ferulic	3.05

### 3.4 Analysis of Pisolithus albus extracts

**Total phenolic and total flavonoid content** Total phenolic and total flavonoids content were measured for the ethanol extract that are displayed in Table 3 and Fig. 4. The former phenol content was found to be 116.6 mg GAE/g, and a total flavonoid content of 165.34 mg quercetin equivalents per g.

### 3.5 HPLC analysis of phenolic and flavonoids compounds

High-performance liquid chromatography (HPLC) was employed to separate and recognize the various individual constituents and compound mixtures present in the ethanol extract of *Pisolithus albu*s. The following Table 4 and Fig. 6 showed the overall chromatography profile obtained over HPLC, and the following separations in fractions for **Flavonoids** showed six fractions, Apegenin was detected at the highest concentration 16.54 μg/m followed by Querestin at 13.95 μg/ml. regarding to **Phenolic compounds** there were also six fraction the highest was Gallic acid (16.75 μg/g) at retention time 9.8, then p-Coumaric 12.52μg/g.

### 3.6 Antioxidant activity

The scavenging action of ethanol extracts was assessed by the antioxidant assays: DPPH method (Table 5). It was applied to evaluate the antioxidant activity of ethanol fungal extract with comparing of ascorbic acid as a standard at different concentrations (1000, 500, 250, 125, 62.5, 31.25, 15.6 and 7.8, 3.9 and 2 μg/ml). The findings indicated that antioxidant activity rose progressively with increasing concentration, demonstrating a clear dose-dependent pattern. The extract showed significant DPPH radical scavenging activities, it showed higher DPPH radical inhibition (94.71%) than standard (98.65%)**.**Under the tested experimental conditions, the extract exhibited antioxidant activity, with an IC_50_ value of 14.17 ± 0.84 μg/ml, whereas the reference standard recorded a lower IC_50_ of 10.21 ± 0.77 μg/ml.

### 3.7 Cytotoxicity evaluation of Pisolithus albus extract

Cytotoxicity activity on three cancer cell lines (HepG-2 human Hepatocellular cancer, MCF-7 human breast cancer, A-549human Lung cancer cells) and two normal human cell line (HSF-1human foreskin fibroblast skin cell and WI-38 diploid human lung fibroblasts were measured and shown in Table 6. CC50 values (presented as three readings) were detected against all cell line using MTT assay under the experimental conditions (24 h treatment). At a concentration of 250 μg/ml, the ethanol extract exhibited cytotoxicity against HepG-2 human hepatocellular carcinoma, MCF-7 breast cancer, and A-549 lung cancer cells, resulting in 88.93%, 79.29%, and 85.02% cell death, respectively. A clear dose-dependent increase in activity was observed with rising concentration. Cytotoxicity on normal cell line was 48.71 and 67.52 for HSF-human foreskin fibroblast skin cell and WI-38 diploid human lung fibroblasts. This study highlights the growing interest in identifying natural, bioactive compounds for cancer therapy. The ethanol extract of the naturally occurring fungus demonstrated strong anticancer effects across all tested cancer cell lines.

### 3.8 Neutron activation analysis (NAA)

Elemental composition of the fungal crude powder was determined by neutron activation analysis (NAA), where samples were first exposed to neutron irradiation and then the resulting induced gamma activity was quantified using a high-purity germanium (HPGe) detector. In our study, the elemental contents of *P. albus* were then determined for two fungal sample from different location using the Neutron activation technique. In the present investigation, eleven elements were identified (Table 7). Among them, K, Na, Ca, and Fe were categorized as major macro-nutrients in both examined samples. Co, Zn, and Cr were detected as trace micronutrients. Apart from that, Br, Sb, Sc and La also were present but their physiological importance is negligible in humans and are considered non-essential to human health. The range of sodium levels recorded was from 461 to 2250 mg/kg. The present study recorded a wide variation in potassium levels, which were the highest among all analyzed elements (16200–31,800 mg/kg). The concentration of calcium was between 2480 and 5260 mg/kg, chromium was found in the range from 11.5 to 22.2 mg/kg, iron level 299 to 761 mg/kg, cobalt was found at low concentrations 0.522–1.41 mg/kg, and zinc was found in the range 45.6–66.4 mg/kg. Some minor elements such as Sc, Sr, Br, and Rb were also found in some samples. The relatively high trace values for Bromine (55.4–80.2 mg/kg) while only trace amounts of the elements Sc, Sb, Cs and La were found in the two samples, and were not regarded as essential, required diligent attention. (See Table 6.)

**Table 5:** Antioxidant activity of ethanol extract of *Pisolithus albus.*

Tested conc. (μg/ml)	DPPH scavenging % Sample extract	DPPH scavenging % standared
1000	94.71 ± 0.57	98.65 ± 0.10
500	91.63 ± 0.19	96.34 ± 0.62
250	85.12 ± 0.64	94.25 ± 0.92
125	79.47 ± 0.65	90.89 ± 0.17
62.5	70.68 ± 0.84	86.42 ± 0.71
31.25	62.49 ± 1.75	79.35 ± 1.22
15.6	53.26 ± 2.32	65.05 ± 0.21
7.8	35.41 ± 1.75	43.01 ± 2.87
3.9	26.89 ± 1.23	30.04 ± 1.04
2	18.43 ± 0.69	22.18 ± 1.37

**Table 7:** Elemental composition of major and trace elements (mg/kg) in the investigated fungal crude powder determined by neutron activation analysis (NAA).

Nuclide	mg/Kg		The recommended daily intake mg/day	safe upper levelUL national research council (NRC)
	Sample 1	Sample 2		
K	16,200 ± 66	31,800 ± 57	4700	no determined
Ca	5260 ± 760	2840 ± 191.5	1300	2500
Na	2250 ± 120	461 ± 140	1500	2300
Fe	761 ± 148	299 ± 57	8–18	45
Zn	45.6 ± 9.9	66.4 ± 7.0	11	40
Co	0.522 ± 0.19	1.41 ± 1.09	0.12	1–2
Cr	11.5 ± 1.16	22.2 ± 7.0	0.035	no determined
Br	55.4 ± 8.8	80.2 ± 3.05	no determined	no determined
Sb	0.0908 ± 0.14	0.00 ± 0.0	no determined	no determined
Sc	0.210 ± 0.07	0.897 ± 0.02	no determined	no determined
La	0.564 ± 0.36	1.59 ± 0.85	no determined	no determined

**Table 6:** Cytotoxicity and antitumor evaluation using viability assayof *Pisolithus albus* ethanolic extract.

Ethanolic extract (μg/ml)	Cytotoxicity %		Inhibitory %		
	HSF-1	WI-38	HepG-2	MCF-7	A-549
500	69.12	87.39	96.05	91.77	93.96
250	48.71	67.52	88.93	79.29	85.02
125	22.08	42.57	73.57	54.03	68.26
62.5	12.05	18.05	50.82	23.76	39.61
31.25	2.80	0.64	32.51	9.35	17.03
15.6	0.31	0	15.49	0.52	1.88
7.8	0	0	2.64	0	0
0 (Non-treated cell control)	0	0	0	0	0
*IC*_*50*_*μg/ml*	265.58 *±* 12.99	*162.22* ± 8.46	*61.10* *±* *2.71*	*116.68* *± 5.04*	*85.17* *±* *3.98*

## 4 Discussion

*Pisolithus albus* is a species is described here for the new record in Egypt, GenBank accession number PX788599. This ectomycorrhizal fungus is usually found with trees in sandy well-drained soils, particularly in coastal pine ecosystems. The literature on this species is still limited, and most of the research has been devoted to the mycorrhizal associations with host plants, genetic diversity, taxonomic classification and capability of improving soil fertility and controlling soil erosion. The antioxidant and antimicrobial activity of bioactive components of *Pisolithus* species is still poorly explored. To investigate their potential as therapeutic candidates, it was decided to evaluate the antioxidant, antimicrobial, and antitumor behavior of fungal extracts in the present work. The antimicrobial activity of *Pisolithus albus* extracts in vitro was assessed using the agar well diffusion method against five different pathogenic bacteria species along with a yeast strain. The ethanol and acetone extracts of *Pisolithus albus* affected *Salmonella typhimurium* and *Bacillus subtilus,* followed by *Ps. aeruginosa* (22 mm), *Bacillus subtilis* (21 mm) and *Staphylococcus aureus* (20.5) by ethyl acetate extract, whereas, the *E. coli* low affected by all tested solvent extracts (15 mm). Regarding to antifungal activity, Ethanol extract affected *Candida albicans* with an inhibition zone (23–18.3 mm) with all tested extracts. The most efficient extract was ethanol extract with MIC 20 mg/ml against all tested pathogenic isolates. It was proven that extracts of ectomycorhazial fungus at a concentration of 10 mg/ml were capable of suppressing the growing of numerous pathogenic bacteria, including *Salmonella typhi coli*, *Escherichia*, *Klebsiella pneumoniae*, and *Pseudomonas aeruginosa*, *Candida albicans* was more sensitive to all tested fungal extract at MIC 20 mg/ml.^8^ Also, The antimicrobial activity of various extracts of *Pisolithus albus* fruiting bodies were tested at 40 mg/ml against 30 MRSA and MSSA strains by Ameri et al.^9^, Their results demonstrated that chloroform extract had no inhibitive activity against any of the bacteria tested while the aqueous extract had weak activity with an inhibition zone of between 7.0 and 11.75 mm. However, the ethyl acetate extract exhibited the greatest antibacterial activity with inhibition zones ranging from 7.0 to 21.25 mm and an average of 14.54 ± 3.23 mm.

Later, Mohan et al.^25^ investigated the antagonistic effects of eight ectomycorrhizal fungi, including *Pisolithus*, in contradiction of nine different plant pathogenic fungi. All tested ECM fungi displayed inhibitory activity; notably, *Pisolithus albus* showed variable suppression levels, with inhibition percentages ranging from 12.22% to 47.78% depending on the target pathogen.

The main antioxidant compounds found in the extracts of the present investigation were total phenolic compounds and total flavonoids. The total phenolic content found was 116.6 mg GAE/g while the total flavonoid content of the ethanol extract was 165.34 mg quercetine/g. Flavonoids are well established in terms of their wide range of biochemical and pharmacological properties, involving antimicrobial, anti- carcinogenic, and antioxidant properties.^26^

High-performance liquid chromatography (HPLC) was further utilized to characterize the bioactive constituents, aiming to separate and identify the various individual compounds and/or compound mixtures present in the ethanol extract of *Pisolithus albus*. Fractions for Flavonoids showed six fractions, Apegenin and Querestin were detected at the highest concentration 16.54 and 13.95 μg/g, respectively. Also, regarding to Phenolic compounds, there were also six fraction, the highest was Gallic (16.75 μg/g) at retention time 9.8, then p-Coumaric 12.52μg/g at RT 6. Multiple studies have documented a variety of bioactive complexes that can be isolated from *Pisolithus tinctorius.*^27^, ^28^, ^29^ Dilsad et al.,^26^ found that the methanolic extracts of mushrooms showed the highest amount of total phenolics and flavonoids, and total antioxidant activity. Furthermore, a high performance liquid chromatography (HPLC) analysis of flavonoids showed that the methanol extracts had relatively higher levels of rutin, luteolin, catechin, and quercetin.

The antioxidant activity of the ethanol extract was evaluated at different concentrations by DPPH assay. The results showed a definite dose-dependent relationship with the radical scavenging activity. The extract showed excellent DPPH inhibition, and was slightly lower than the standard compound (98.65%). The findings are in agreement with Sevindik, et al.,^30^ who found *P. arrhizus* have good antioxidant activity. Accordingly, it is suggested that the bioactive constituents responsible for the antioxidant activity of *P. arhizus* could be isolated and potentially utilized as natural antioxidant agents.

Cytotoxicity activity on three cancer cell lines (HepG-2 human Hepatocellular cancer, MCF-7 human breast cancer, A-549 human Lung cancer cells) and two normal human cell line (HSF-human foreskin fibroblast skin cell and WI-38 diploid human lung fibroblasts were measured, a concentration of 250 μg/ml, the ethanol extract induced marked cytotoxicity, leading to the death of 88.93% of HepG-2 carcinoma cells, 79.29% of MCF-7 human breast cancer cells, and 85.02% of A-549 human lung cancer cells. A dose-dependent response was observed, where the level of cell mortality increased progressively with rising extract concentrations. The cytotoxicity of normal cell line was 48.71 and 67.52 for HSF-human foreskin fibroblast skin cell and WI-38 diploid human lung fibroblasts, respectively. The demand for identifying effective natural compounds in cancer therapy has been increasingly emphasized. In the present work, the investigated naturally derived fungus exposed strong anticancer activity versus all tested cancer cell lines. Measuring the cytotoxic effects, the 3-(4,5-dimethylthiazol-2-yl)-2,5-diphenyl tetrazolium bromide (MTT) assay was used in liver cells, and basidiomycetous mushrooms were reported to be with a remarkable anticancer potential.^31^ Alves et al.,^28^ **c**ompared extracts extracted from spores of *Pisolithus tinctorius* using dichloromethane–methanol (2:1) and EtOAc–MeOH (2:1), revealing that the dichloromethane–methanol (2:1) extract had the greatest cytotoxic effect against cancer cell lines. Dilsad et al.,^26^ have also demonstrated significant cytotoxicity of ethanol and methanol extracts of *P. arrhizus* in colon cancer cells in a concentration-dependent manner.

Heavy metals are very poisonous and can cause a progressive damaging effect over time to various organs. They are known to have a vascular toxicity linked to several pathological disorders, particularly due to their ability to increase oxidative stress, inflammation and coagulation activity. In this aspect, it was recommended that *P. albus* could be used as a significant source of antioxidant compounds. The elements that enter the human body are divided into two categories, essential and non-essential elements, depending on the physiology that they serve.^32^, ^33^ Essential elements incorporate N, K, P, Ca, Cl, Fe, Mn, Mg, Zn, Ni, Co, Cu, Na, Se, Cr and many others. These are also distinguished into macro- and microelements according to their occurrence in biological systems. Macro-elements—these are present in relatively large quantities (N, K, P, Mg, Ca, Na, Cl and Fe). They are essential for maintaining a number of important physiological processes such as bone health, dental health, blood health, protein synthesis, energy metabolism, cell growth, cardiovascular function, and nervous system function.^34^ In this present study, quantitative analysis of a total of 11 elements was carried out. Of these, Ca, K, Na and Fe were detected as macro-nutrients in both samples analyzed, while Co, Zn and Cr were classified as trace (micro) essential elements. Additionally Br, Sb, Sc and La were detected, but are believed to be non-essential for human health because they do not have physiological functions. The sodium level varied from 461 to 2250 mg/kg. According to the US NRC, the recommended daily dose (RDI) of Na is 1500 mg/day, and the tolerable upper intake level (UL) is 2300 mg/day. Potassium was the most abundant mineral with levels ranging from 16,200 to 31,800 mg/kg, and an RDI of about 4700 mg/day, but no UL has been established. Calcium content varied from 2480 to 5260 mg/kg; its RDI is 1300 mg/day and the UL is 2500 mg/day (US NRC). Concentrations of chromium were measured at 11.5–22.2 mg/kg and there is no RDI or UL. Iron range was 299–761 mg/kg; the RDI for men is 8 mg/day and for women 18 mg/day, and the UL is approximately 45 mg/day. The contents of cobalt ranged from 0.522 mg/kg to 1.41 mg/kg, the RDI is 0.12 mg/day, and the UL is 1–2 mg/day. The concentrations of zinc were in the range of 45.6–66.4 mg/kg, RDI-11 mg/day, UL-40 mg/day. Non-essential elements, such as Sc, Sb, Cs and La were also found at low levels throughout the samples, with bromine reaching 55.4–80.2 mg/kg. Other researchers had previously used atomic absorption spectrophotometry (AAS) to determine elemental composition of mushrooms, and it was observed that the heavy metal content of *P. arhizus* was generally safe.^30^ Similarly, Naveen and Raja^36^ determined the concentration of heavy metals (Ni, Pb, Cr, Cd) in AAS and it was found that the highest concentration was for chromium (13.25%) and followed by Ni (2.35%).

## 5 Conclusion

Fungi are well known for their medicinal properties, comprising their antioxidant, antibacterial, antifungal and anticancer effects, especially the macro-fungi that produce bioactive metabolites. From this point of view, *Pisolithus* albus is an interesting species for study, because of the number of its chemical and pharmacological properties that has not been well documented in studies of its fruiting bodies. The present morphological and molecular identification confirmed that *Pisolithus albus* was a new record Egypt and the collected specimens were identical with the data of GenBank accession number (PX788599). The antimicrobial properties of *P. albus* extracts in ethanol were found to be significant against pathogenic bacteria and yeast. Chromatographic methods based on HPLC were used to obtain the highest extraction efficiency of bioactive constituents. *P. albus* also showed high antioxidant properties and the compounds present in it might be isolated and used as natural antioxidant agents. Dose-dependent activity was seen in the cytotoxic assays against three cancer cell lines (HepG-2 hepatocellular carcinoma, MCF-7 breast cancer and A-549 lung cancer), as well as 2 normal human cell lines (HSF foreskin fibroblasts and WI-38 lung fibroblasts) with higher concentrations of extract yielding higher degrees of inhibition. The cytotoxicity values for normal cells were 48.71% and 67.52% for HSF and WI-38, respectively. The neutron activation analysis results for *P. albus* showed generally acceptable concentrations of heavy metals, with relatively high concentrations of iron. The overall results indicate that *P. albus* is a potential source of bioactive compounds. Future applications can be related to its metabolites, which can be used for the improvement of pharmaceutical agents with anticancer, antibacterial, antifungal and antioxidant properties, as a multifunctional natural drug candidate.

## CRediT authorship contribution statement

**Sahar M. Ouda:** Writing – review & editing, Writing – original draft, Methodology, Funding acquisition, Formal analysis. **Mohamed G.A.:** Writing – review & editing, Writing – original draft, Methodology, Funding acquisition, Formal analysis.

## Funding

No funding received.

## Declaration of competing interest

The authors declare that they have no known competing financial interests or personal relationships that could have appeared to influence the work reported in this paper.
